# Influence of odontogenic lesions on root development in impacted teeth: a cohort study

**DOI:** 10.3389/froh.2025.1634188

**Published:** 2025-08-18

**Authors:** Selene Barone, Alessandro Antonelli, Antonio Madonna, Vincenzo Greco, Massimo Borelli, Francesco Bennardo, Amerigo Giudice, Lucia Cevidanes

**Affiliations:** ^1^ Department of Health Sciences, Magna Graecia University of Catanzaro, Catanzaro, Italy; ^2^ Department of Orthodontics and Pediatric Dentistry, University of Michigan, Ann Arbor, MI, United States

**Keywords:** impacted teeth, odontogenic lesion, root dilaceration, marsupialization, dental anomalies

## Abstract

**Introduction:**

Root dilaceration, a clinically significant developmental anomaly that can complicate dental treatment, has been attributed to various etiological factors, but the role of odontogenic lesions is still poorly understood. This observational study aimed to evaluate the relationship between odontogenic lesions and root dilaceration in impacted teeth.

**Methods:**

The sample size consisted of 22 impacted teeth divided into two groups: with odontogenic lesions (Group IwL) and without lesions (Group IwoL). Pre- and post-treatment radiographs, taken before and after conservative surgical or orthodontic-surgical management of impacted teeth, were used to assess the occurrence of dilaceration in both groups. Fisher's exact text was applied to compare the prevalence of dilaceration in both groups. In order to analyze the influence of each additional variable on dilaceration, a multivariate analysis was performed through logistic regression.

**Results:**

Root dilaceration was significantly more common in Group IwL (72.73%) than in Group IwoL (18.18%) (*p* = 0.030). No significant association was found between root dilaceration and additional variables, including impaction depth, cortical bone contact, maximum lesion size, and lesion volume.

**Conclusions:**

This study provides novel evidence for a correlation between odontogenic lesions and root dilaceration, suggesting that compressive forces from these lesions may significantly contribute to abnormal root development, with important implications for clinical diagnosis and treatment planning.

## 1 Introduction

Eruption anomalies, including impacted teeth, are common in dental practice. Impacted teeth fail to erupt into the oral cavity within the expected developmental timeframe. This condition is frequently observed in the third molars (wisdom teeth), maxillary canines, and premolars, though it can affect any tooth (1, 2). The prevalence of impacted teeth varies by population and is influenced by genetic, environmental, and anatomical factors (3). Studies estimate that approximately 10% of individuals experience some form of dental impaction, with molars and canines being the most affected due to spatial constraints in the dental arch (4). The etiology of tooth impaction is multifactorial. It can be due to physical barriers such as adjacent teeth, dense bone, pathological lesions, soft tissue obstruction, or misalignment of the tooth's eruption path (3, 5–8). Genetic predispositions, such as cleidocranial dysplasia and other craniofacial anomalies, are significantly related to tooth impaction. In many cases, impacted teeth remain asymptomatic and undetected until radiographic detection or complications arise (3).

Odontogenic lesions like cysts or tumors are often associated with impacted teeth. These lesions can arise from the dental follicle, the developmental structure surrounding an unerupted tooth (9). Such lesions can exert compressive forces on the developing tooth, potentially influencing its root formation (10). One of the root developmental anomalies observed in association with these lesions is root dilaceration, an abnormal bending or curving of the tooth root (11, 12). This condition may complicate the surgical orthodontic treatment of the impacted tooth and affect its long-term prognosis (13).

Given the potential complications associated with odontogenic lesions and their impact on root development, it is important to investigate the relationship between these lesions and impacted teeth development. While previous studies have explored various causes of root dilaceration and its association with dental trauma, the specific role that odontogenic lesions play in this process remains unknown (14). Understanding this relationship is critical for clinicians to make correct decisions regarding the timing and method of intervention for impacted teeth.

This observational retrospective cohort study aims to evaluate whether an odontogenic lesion associated with impacted teeth affects their root development. Specifically, this study investigated the association between odontogenic lesions and root dilaceration by comparing the radiographic occurrence of this anomaly in impacted teeth with and without associated lesions, both before and after surgical intervention. Elucidating this relationship may improve understanding of the etiological factors contributing to this clinically challenging anomaly and inform preoperative assessment and treatment planning for impacted teeth.

## 2 Materials and methods

### 2.1 Study design

This study was designed as an observational retrospective cohort study. The ethical principles followed the Declaration of Helsinki for the protection of human subjects in medical research. The regional Ethical Review Board of Central Calabria (reference for the Magna Graecia University of Catanzaro) approved the study (n.146/2025).

### 2.2 Study sample

All patients provided signed informed consent for using their data for research purposes. Radiographs obtained from the database of the Unit of Oral Surgery and Pathology of Magna Graecia University of Catanzaro were reviewed, and a total of 21 patients were consequently enrolled with the following characteristics: growing patients with impacted teeth and incomplete root development; no history of dentofacial trauma; patients who underwent either conservative surgical (marsupialization) or orthodontic-surgical treatment using a skeletal anchorage device to recover the impacted teeth; good general health. The included patients were divided into two groups concerning an odontogenic lesion associated with an impacted tooth.

The exclusion criteria were as follows: history of previous surgery for impacted teeth; presence of craniofacial syndromes or congenital anomalies; poor quality radiographic images.

*a priori* power analysis was performed using G*Power 3.1 software (15), based on an expected dilaceration prevalence of 80% in the group with odontogenic lesion-associated impacted teeth and 20% in the group without associated lesions (*α* = 0.05; power = 0.8), indicating a required sample size of at least 10 teeth per group.

### 2.3 Assessment of radiological data

A preoperative cone beam computed tomography (CBCT; X-Mind® Trium, Acteon®, Mérignac, FR) scan was acquired from the database, as it is crucial for planning the surgical treatment of impacted teeth. At the 1-year follow-up, a postoperative panoramic radiograph or a CBCT scan was performed, depending on the severity of the impaction or the associated lesion, to evaluate the eruption of the impacted teeth and the healing of the surrounding tissues. The radiographs were anonymized and independently reviewed by three authors (SB, AM, and VG) using a qualitative assessment to evaluate root development by comparing postoperative and preoperative findings in the two groups. Any discrepancies were discussed and resolved in consultation with an expert author (AG). Supplementary radiographic evaluations were carried out for each impacted tooth associated with lesions to assess the depth of impaction, the contact with the cortical bone for the teeth, the maximum size in each anatomical plane, and the volume of the lesions.

### 2.4 Three-dimensional processing and analysis

After data anonymization, each CBCT was automatically oriented in the software 3DSlicer (version 5.8.1; http://www.slicer.org), using the Automatic Standard Orientation (ASO) tool, according to the Frankfurt and median sagittal planes (16). Automated landmark identification was performed to measure the additional variables (17). The most coronal point of the impacted tooth was identified, and the occlusal plane was traced in the sagittal view. The two outermost points of the lesion in the axial, sagittal, and coronal planes were identified.

Finally, the lesion was three-dimensionally reconstructed to calculate its volume.

### 2.5 Study variables

The primary predictor variable was the presence or absence of an odontogenic lesion associated with the impacted tooth, with patients divided into those with a lesion (Group IwL) and those without a lesion (Group IwoL).

The primary outcome variable assessed in this study was root development analysis, considering the prevalence of root dilaceration in Group IwL and Group IwoL. This was identified qualitatively based on the presence of any abnormal curvature or angulation of the root in the postoperative images compared to the preoperative images.

**Figure 1:**
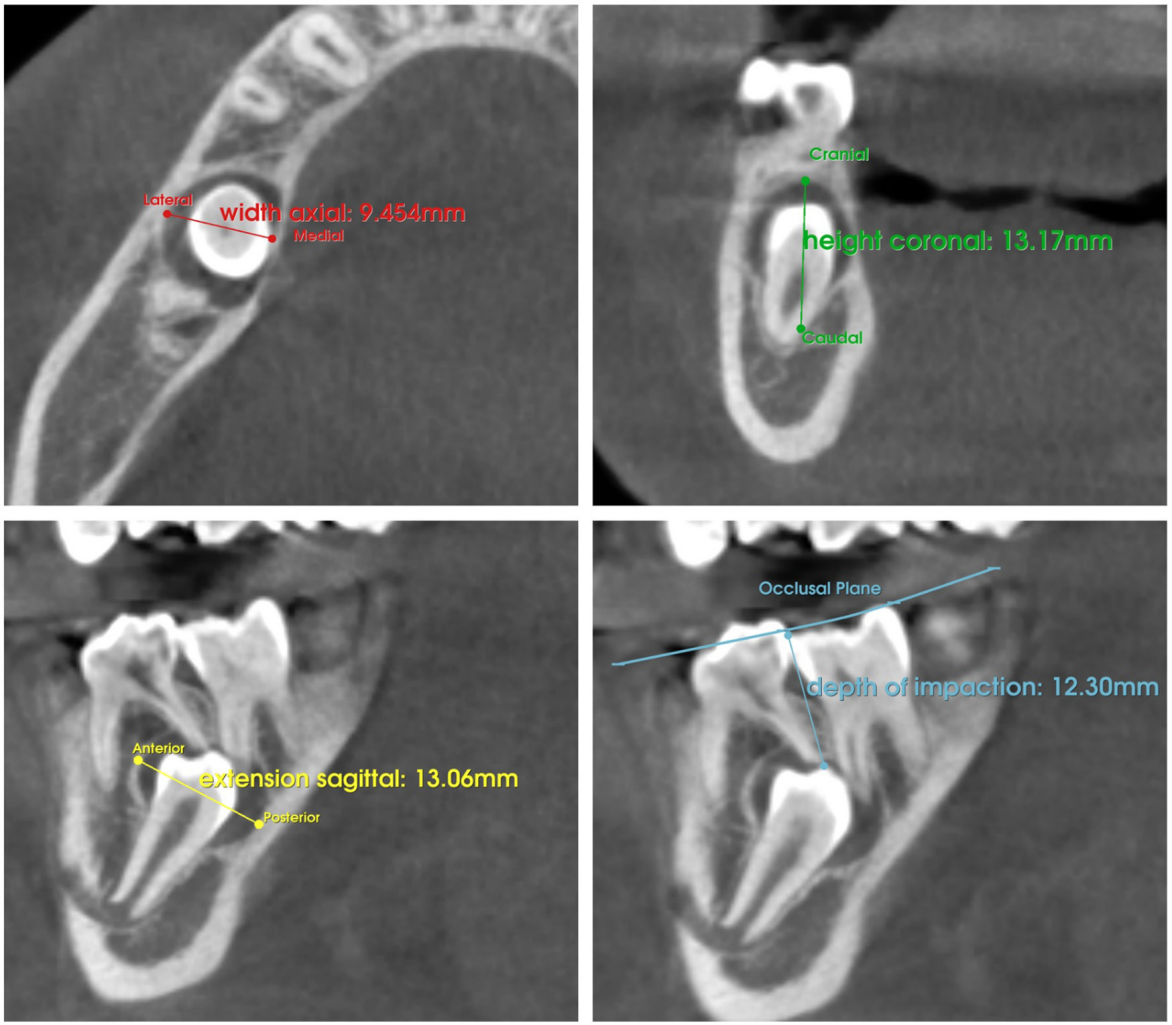
Automatic assessment of lesion size on axial, coronal, and sagittal planes and the depth of tooth impaction following landmark identification.

Additional variables included the depth of tooth impaction, the contact of the tooth with the cortical bone, the maximum lesion size, and the lesion volume. The depth of tooth impaction was automatically measured from the most coronal landmark of the crown to the occlusal plane (Figure 1). The contact between the impacted tooth and cortical bone was assessed to verify the presence or absence of continuity between them. The maximum lesion size was automatically measured between the two outermost points in each anatomical plane (Figure 1). The lesion volume was calculated in cubic millimeters based on 3D segmentation.

### 2.6 Statistical analysis

Recorded data were collected in an Excel file (version 16.85, Microsoft, Redmond, WA, USA). The statistical analyses were conducted using R Studio software (version 4.3.0, 250 Northern Ave, Suite 420, Boston, MA, USA, 02210). Descriptive analyses were performed to obtain the mean and standard deviation for continuous quantitative variables, and frequencies and percentages for categorical data. A Fisher's exact test was performed to compare the prevalence of root dilaceration between groups IwL and IwoL. A multivariate logistic regression analysis was conducted to assess the association between root dilaceration and additional variables. The significance level was set at *α* ≤ 0.05.

## 3 Results

Between March 2020 and September 2023, twenty-five patients were recruited for this study. Three patients were excluded because they did not meet the inclusion criteria. Twenty-one patients with a mean age of 12 ± 2.6 years (13F, 8M) were finally included in the study sample for 22 impacted teeth (Table 1), with one patient presenting two impacted teeth. Group IwL consisted of 11 impacted teeth, and Group IwoL included 11 impacted teeth. Demographic variables were reported in Table 1.

**Table 1:** Descriptive statistics of the study sample.

Demographic variables	Study Sample	Group IwL (dilacerated)	Group IwoL (dilacerated)
Patients *n*	21	11	10
Age (years)	12 ± 2.6 years	10.3 ± 1.9 years	13.7 ± 2.1 years
Sex			
Female	13	7 (4)	6 (2)
Male	8	4 (4)	4 (0)
Ethnicity			
Caucasian	20	10 (7)	10 (2)
Asian	1	1 (1)	0
Impacted teeth *n*	22	11 (8)	11 (2)
Incisor	3	3 (1)	0
Canine	8	1 (1)	7 (1)
Premolar	3	1 (1)	2 (1)
Molar	8	6 (5)	2 (0)
Non-inflammatory cysts *n*	7		
Dentigerous cyst	6	6 (5)	—
Sialo-odontogenic cyst	1	1 (0)	—
Odontogenic tumors *n*	4		
Odontoma	4	4 (3)	—

Continuous data are reported as mean ± standard deviation (SD), and categorical variables are presented as numbers (*n*).

Among the odontogenic lesions associated with impacted teeth, non-inflammatory cysts were found in 7 cases, while benign odontogenic tumors were observed in the remaining 4 cases. Dentigerous cysts were the most common, followed by odontoma (Table 1).

**Figure 2:**
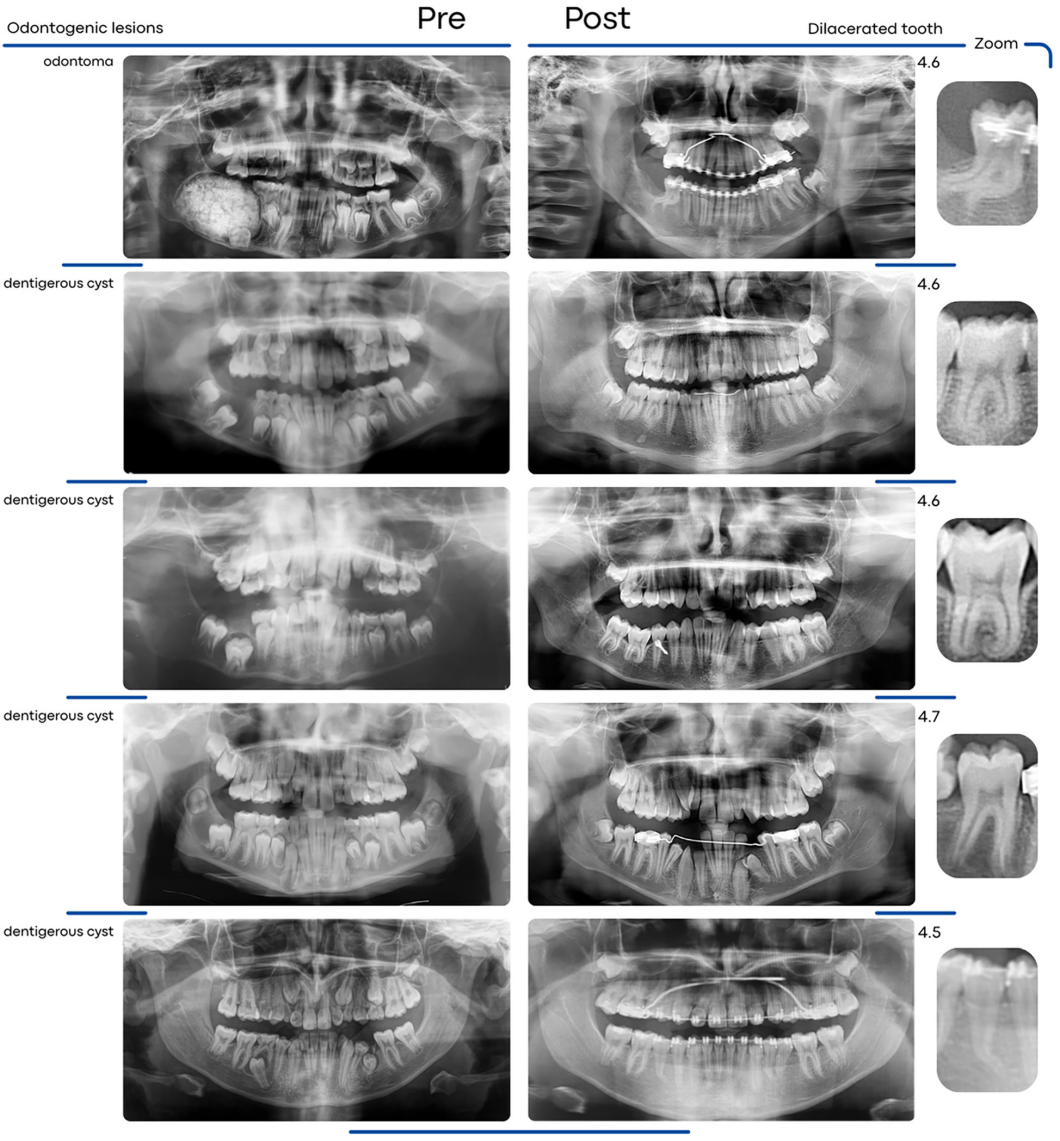
Pre- and post-treatment panoramic radiographs illustrating the development of root dilaceration following surgical-orthodontic management in impacted teeth associated with odontogenic lesions.

Ten dental dilacerations were observed. The study found that root dilaceration was significantly more frequent in Group IwL (Figure 2), with 8 cases (72.73%), compared to Group IwoL, which had only 2 cases (18.18%). This difference was statistically significant (*p* = 0.030). The observed dilaceration prevalences were not significantly different from the expected ones (*p* = 0.82 and *p* = 1, respectively).

Dilacerated teeth were predominantly observed in the posterior region (five molars and two premolars) than in the anterior region (one incisor and two canines).

In Group IwL, no statistically significant association was found between root dilaceration and the depth of tooth impaction, tooth contact with cortical bone (buccal cortical, lingual cortical, or inferior mandibular margin), maximum lesion size (width axial, extension sagittal, height coronal), and lesion volume (Table 2).

**Table 2:** Measurements of additional variables analyzed in group IwL.

Additional variables	Min	Max	Yes (dilacerated)	*p*-value^a^
Impaction depth	3.3 mm	22.46 mm	—	0.999
Cortical impaction	—	—	7 (5)	0.999
Width axial	7.8 mm	33.39 mm	—	1.000
Extension sagittal	9.8 mm	41.49 mm	—	1.000
Height coronal	12 mm	35.14 mm	—	0.999
Lesion volume	345 mm^3^	16,900 mm^3^	—	0.999

^a^ The association between tooth dilaceration and additional variables was addressed by the logistic regression.

*p* ≤ 0.05 considered statistically significant.

## 4 Discussion

This study examined the effect of odontogenic lesions associated with impacted teeth on root development, specifically root dilaceration. Dilaceration, defined as a sharp deviation of the tooth's long axis in the root portion, poses significant challenges for diagnostic assessment and treatment planning. While some authors consider dilaceration present when the root deviates by 20 degrees or more from the tooth's axial direction (18), there is still considerable variability in its definition and reported prevalence. While case reports have documented instances of root dilaceration in teeth associated with odontogenic lesions (9, 19–21), to our knowledge, no previous studies have systematically investigated the potential etiological role of these lesions in developing this anomaly. By comparing radiographic data from impacted teeth with and without associated lesions, both before and after treatment, this study sought to provide new insights into the developmental sequelae of these pathologies.

**Figure 3:**
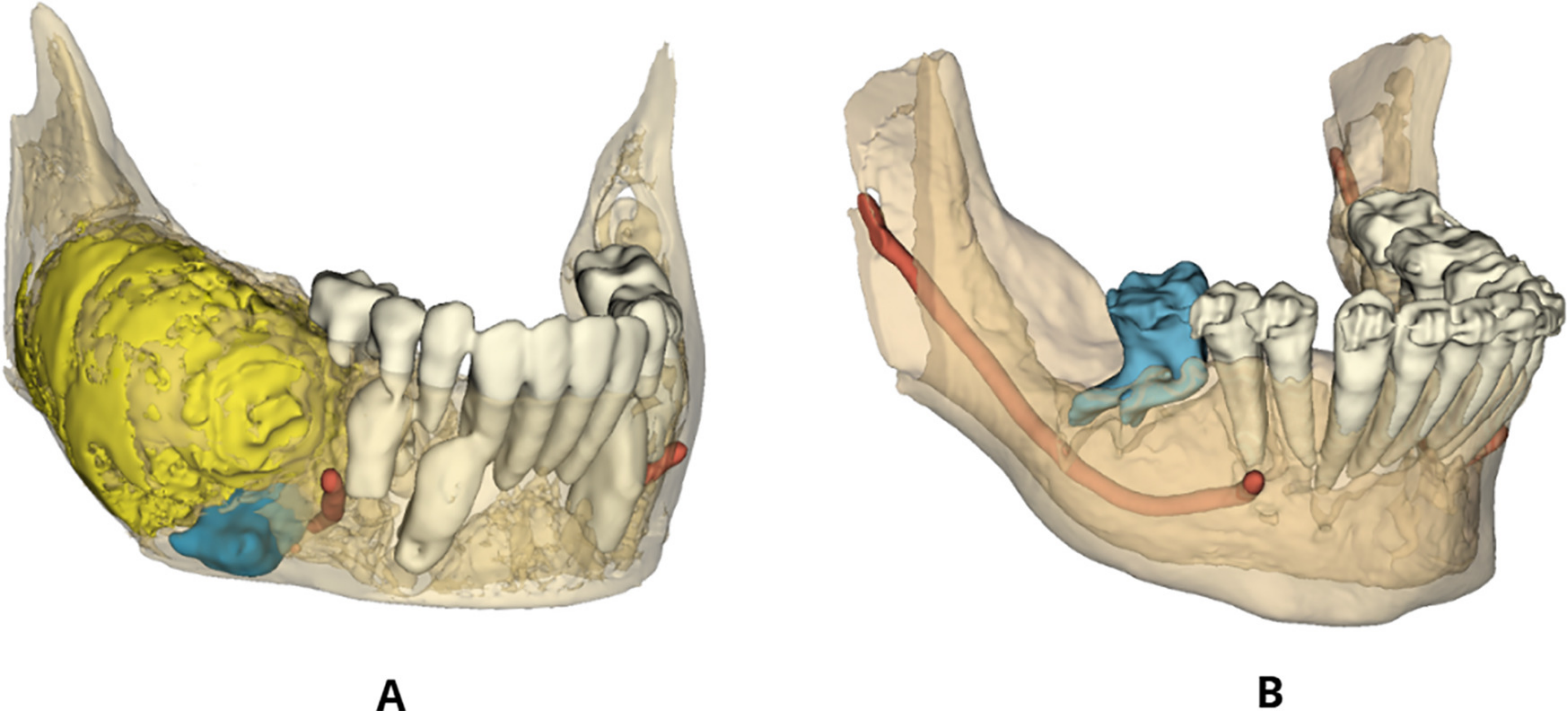
Three-dimensional segmentation showing the development of root dilaceration in Group IwL. **(A)** 3D reconstruction of an odontogenic lesion-associated impacted tooth before surgical-orthodontic treatment. **(B)** 3D reconstruction of a dilacerated tooth after treatment.

The two groups had significantly different prevalences of root dilaceration following surgical or orthodontic surgical treatment, suggesting a possible correlation between the presence of odontogenic lesions and root dilaceration in impacted teeth (Figure 3).

A permanent tooth may present with a dilaceration asymptomatically or with an extended retained primary tooth, failure of the dilacerated tooth to erupt, or fenestration of the cortical bone (9). Identifying dilaceration is essential, as irreversible root resorption and cortical bone perforation have been documented in dilacerated teeth that have undergone orthodontic treatment (13, 20, 21). This aspect is crucial in evaluating the tooth extraction as a single surgical treatment as an alternative to the orthodontic-surgical approach (13). Moreover, the presence of an odontogenic lesion requires a greater focus on managing dilacerated teeth when evaluating treatment options. The combination of marsupialization to decompress the cystic lesion and orthodontic traction can recover severely impacted and dilacerated teeth associated with odontogenic lesions (20). Indeed, by reducing intracystic pressure, marsupialization creates a more favorable environment for the eruption of odontogenic lesion-associated impacted teeth with an open apex or incomplete root development (22, 23).

The etiology of dilacerations has not been fully defined; however, two leading causes have been reported in the literature: trauma to primary teeth and idiopathic developmental disorders of the dental germ (24).

The first hypothesis, which is the most accepted, is that acute mechanical trauma to the primary tooth can cause displacement of the dental germ of the underlying permanent tooth, resulting in dilaceration (25). However, the incidence of dilaceration in permanent teeth is low compared to primary teeth, suggesting that trauma may not be the only etiological factor (18, 26).

The second hypothesis proposes that dilaceration is caused by idiopathic disturbances in the development of the dental germ, especially in cases with no clear history of trauma (25, 27). This theory is supported by the fact that dilaceration is more commonly observed in the posterior teeth, which are less susceptible to direct trauma. In some cases, trauma may not be recognized or remembered due to early childhood injuries that parents may not consider significant (18, 28, 29). All these considerations make it necessary to investigate the potential presence of additional causes. Although rarely reported in the literature, an odontogenic lesion can be considered a cause of dilaceration. The physical force of the lesion appears to affect root growth, resulting in a change in the angulation of the impacted tooth (9). The impact of lesion pressure on root development is probably the one that potentially leads to dilaceration.

The available literature has documented cases of root dilaceration in impacted teeth associated with odontogenic lesions. In 1997, Dayi et al. (19) highlighted the possible relationship between odontogenic lesions and root dilaceration by describing an odontogenic adenomatoid tumor associated with a dilacerated lateral incisor. More recently, Yeung et al. (9) reported a case of an odontoma-associated primary tooth with root dilaceration. Consistent with these previous studies, Abu-Mostafa et al. (20) described a case report of an impacted and dilacerated premolar surrounded by a dentigerous cyst.

In 2022, Enache et al. (21) reported five cases of dentigerous cysts associated with root dilacerations, suggesting that there may be a direct correlation between odontogenic lesions and dilacerations in the simultaneous presence of genetic or local factors.

This comparative study provides preliminary evidence suggesting an association between odontogenic lesions and root dilaceration in impacted teeth. The statistically significant difference in dilaceration prevalence between those with and without associated lesions indicates that the compressive forces exerted by these lesions may negatively influence root development, but further research is needed to confirm this potential causal relationship. Even though this study does not directly demonstrate that compressive forces from the lesions cause the dilaceration, the possible mechanistic role of compressive forces is due to the known expansile nature of odontogenic lesions, the plausibility that physical pressure on the developing root could alter its direction of growth, and prior case reports and series noting dilaceration of impacted teeth in the presence of odontogenic cysts and tumors (9, 19–21).

In addition, although cortical impaction was not statistically significant, contact between the impacted tooth and adjacent cortical structures, including the buccal, lingual cortical, and inferior mandibular margin, was frequently observed in cases with root dilaceration. Notably, the degree of root curvature appeared to be more pronounced when such contact was present.

The present study findings emphasize the need for additional research in larger cohorts with a multicentric design to extend our understanding of whether lesion size and tooth impaction severity influence root development. Future research should consider incorporating a specific assessment of the orthodontic treatment difficulty according to the degree of root dilaceration. Such results have potential implications for clinical treatment, as early detection and management of such lesions may help prevent complications in root formation.

## Data Availability

The raw data supporting the conclusions of this article will be made available by the authors, without undue reservation.
